# *Pseudomonas aeruginosa* Exhibits Deficient Biofilm Formation in the Absence of Class II and III Ribonucleotide Reductases Due to Hindered Anaerobic Growth

**DOI:** 10.3389/fmicb.2016.00688

**Published:** 2016-05-09

**Authors:** Anna Crespo, Lucas Pedraz, Josep Astola, Eduard Torrents

**Affiliations:** Bacterial Infections and Antimicrobial Therapies, Institute for Bioengineering of CataloniaBarcelona, Spain

**Keywords:** ribonucleotide reductases, DNA synthesis, *Pseudomonas aeruginosa*, biofilm formation, anaerobic metabolism, oxygen diffusion, *nrd* genes, vitamin B_12_

## Abstract

Chronic lung infections by the ubiquitous and extremely adaptable opportunistic pathogen *Pseudomonas aeruginosa* correlate with the formation of a biofilm, where bacteria grow in association with an extracellular matrix and display a wide range of changes in gene expression and metabolism. This leads to increased resistance to physical stress and antibiotic therapies, while enhancing cell-to-cell communication. Oxygen diffusion through the complex biofilm structure generates an oxygen concentration gradient, leading to the appearance of anaerobic microenvironments. Ribonucleotide reductases (RNRs) are a family of highly sophisticated enzymes responsible for the synthesis of the deoxyribonucleotides, and they constitute the only *de novo* pathway for the formation of the building blocks needed for DNA synthesis and repair. *P. aeruginosa* is one of the few bacteria encoding all three known RNR classes (Ia, II, and III). Class Ia RNRs are oxygen dependent, class II are oxygen independent, and class III are oxygen sensitive. A tight control of RNR activity is essential for anaerobic growth and therefore for biofilm development. In this work we explored the role of the different RNR classes in biofilm formation under aerobic and anaerobic initial conditions and using static and continuous-flow biofilm models. We demonstrated the importance of class II and III RNR for proper cell division in biofilm development and maturation. We also determined that these classes are transcriptionally induced during biofilm formation and under anaerobic conditions. The molecular mechanism of their anaerobic regulation was also studied, finding that the Anr/Dnr system is responsible for class II RNR induction. These data can be integrated with previous knowledge about biofilms in a model where these structures are understood as a set of layers determined by oxygen concentration and contain cells with different RNR expression profiles, bringing us a step closer to the understanding of this complex growth pattern, essential for *P. aeruginosa* chronic infections.

## Introduction

*Pseudomonas aeruginosa* is a common Gram-negative bacterium that is recognized for its ubiquity and its advanced antibiotic resistance mechanisms. It is also relevant for its great adaptability, being able to inhabit many different environments; it can live free in soil and water and can grow in human and plant host-associated environments. This bacterium is related to clinically relevant human infections in immunocompromised patients and other risk groups. In particular, it causes severe chronic lung infections in patients suffering from cystic fibrosis (CF) or chronic obstructive pulmonary disease (COPD; Lyczak et al., 2002; Davies et al., 2007; Ito and Barnes, 2009).

The establishment of chronic *P. aeruginosa* infections correlates with the formation of biofilm, a structure with clusters of cells encapsulated in a complex extracellular polymeric matrix. Bacteria in biofilms display different patterns of gene expression and phenotypes, reducing their metabolic rate and increasing cell-to-cell communication (Costerton et al., 1999) while becoming less sensitive to chemical and physical stresses, and they show increased chances of developing new antibiotic resistances (Xu et al., 1998; Stewart and Franklin, 2008). Oxygen does not diffuse freely through the biofilm structure, leading to the formation of an oxygen concentration gradient, which generates anaerobic microenvironments (Xu et al., 1998; Werner et al., 2004; Stewart and Franklin, 2008). The oxygen (and other chemical compounds) gradients are major driving forces for regulating the morphogenesis of the biofilm (Dietrich et al., 2013; Kempes et al., 2014; Okegbe et al., 2014).

While usually listed as an obligate aerobe, *P. aeruginosa* is able to grow in the absence of oxygen via anaerobic respiration using nitrates or other oxidized forms of nitrogen (NO_2_, NO) as electron acceptors in a chain of reductions ending in molecular nitrogen (N_2_; Schobert and Jahn, 2010; Arat et al., 2015). The Anr, Dnr, and NarL transcriptional factors are essential for regulating the expression of genes that encode the enzymes needed for denitrification, as well as regulating other genes related to anaerobic metabolism (Schreiber et al., 2007; Arai, 2011). Anr acts as a global oxygen-sensing regulator, controlling essential enzymes such as arginine deiminase and nitrate reductase and controlling *dnr* and *narL* gene expression. Dnr is a NO sensor and is able to modulate the expression of several genes under anaerobic conditions, including the enzymes thought to be involved in dissimilatory nitrogen reduction. NarL is a member of the NarLX two-component system, also thought to be involved in the regulation of nitrate reduction (Benkert et al., 2008). Bioinformatic studies have failed to identify differences between the Anr and Dnr binding sites (Trunk et al., 2010).

Anaerobic growth in *P. aeruginosa* biofilms is thought to be essential for full biofilm establishment (Stewart and Franklin, 2008) and has proven to be clinically relevant. In chronic CF lung infections, it has been shown that *P. aeruginosa* grows in low-oxygen environments within mucus plugs or biofilms (Schobert and Jahn, 2010). Furthermore, it has been shown that microaerophilic and anaerobic conditions are predominant in the sputum of patients with CF (Yoon et al., 2002; Alvarez-Ortega and Harwood, 2007; Hassett et al., 2009).

As another manifestation of its metabolic versatility, *P. aeruginosa* is one of the few microorganisms that encodes the three different ribonucleotide reductase classes in its genome. ribonucleotide reductases (RNRs) are key enzymes that catalyze the reduction of all four ribonucleotides to their corresponding deoxyribonucleotides, providing the necessary precursor molecules for DNA synthesis and repair in all organisms (Cotruvo and Stubbe, 2011; Sjoberg and Torrents, 2011; Hofer et al., 2012; Torrents, 2014; Lundin et al., 2015). RNRs are divided into three classes (I, II, and III) based on their structural differences, metallocofactor requirements, and the mechanisms used for radical generation. Class I RNRs require oxygen to produce a tyrosyl radical using a diferric iron or a dimanganese iron center and, thereby, function only under aerobic conditions. Class II RNRs require adenosylcobalamin (AdoCob) for radical generation and do not depend on oxygen (Torrents et al., 2005; Sjoberg and Torrents, 2011). Class III RNR belongs to the family of glycyl radical enzymes. The radical is generated by an activating enzyme with a (4Fe-4S) cluster that catalyzes the reduction of *S*-adenosylmethionine (SAM). This class can only function under anaerobic conditions. Genes for active representatives of all three classes are present in *P. aeruginosa* metabolism: class I, subclass Ia (*nrdAB*), class II (*nrdJab*), and class III (*nrdDG*). Exceptionally the *P. aeruginosa* class II RNR is splitted and expressed in two different polypeptides (denoted as *nrdJa* and *nrdJb*; Torrents et al., 2005; Crona et al., 2015). The presence and coordinated activity of the three classes is essential to ensure a supply of precursor molecules for DNA synthesis under both aerobic and anaerobic conditions (Sjoberg and Torrents, 2011). However, specifically in *P. aeruginosa* the synthesis of vitamin B_12_ only occurs in aerobic conditions (Lee et al., 2012) and its availability determines the class II RNR activity. Unfortunately, the exact role of each class and how they are genetically regulated is not yet fully understood.

In this work we aimed to study the importance of the different *P. aeruginosa* RNR classes for biofilm formation. We assessed the effect of class II and class III RNR deletion on static and continuous-flow biofilm formation and examined the phenotypic effects of this inactivation to establish the essential roles of RNRs in proper biofilm development. We also studied the genetic regulation responsible for modulating class II and class III RNR gene expression in biofilms, and we incorporated our data into a model where the *P. aeruginosa* biofilm is considered a set of layers determined by oxygen concentration gradients, vitamin-B_12_ and cells with different RNR expression profiles.

## Materials and Methods

### Bacterial Strains, Plasmids, and Growth Conditions

All bacterial strains and plasmids are listed in **Supplementary Table S1**. *Escherichia coli* and *P. aeruginosa* cells were routinely grown in Luria-Bertani broth (LB) at 37°C. Anaerobic growth occurred in LB medium containing KNO_3_ (10 g/l; LBN medium) or 1 mM *S*-nitrosoglutathione (GSNO) in screw-cap tubes (Hungate Tubes) that were purged with N_2_ (Garriga et al., 1996; Arai, 2003). For the anaerobic culture of *P. aeruginosa anr, dnr*, and *narL* isogenic mutant strains, which are not able to grow anaerobically, cells were first grown under aerobic conditions in LB medium to a mid-exponential phase (OD_550_ = 0.5) and then the cultures were pelleted, resuspended in the same volume of LBN medium, and inoculated into screw-cap tubes containing anaerobic LBN medium. Finally, they were incubated for 3 h to induce anaerobic metabolism.

When necessary, antibiotics were added at the following concentrations: for *E. coli*, 10 μg/ml gentamicin and 50 μg/ml ampicillin and for *P. aeruginosa*, 150 μg/ml gentamicin, 300 μg/ml carbenicillin and 50 μg/ml tetracycline. Vitamin B_12_ was added when necessary at a concentration of 1 μg/mL.

### DNA Manipulations and Construction of Plasmids and Strains

Recombinant DNA techniques were performed using standard procedures (Sambrook et al., 1989). DNA fragments were amplified via PCR using High-Fidelity PCR Enzyme Mix (Fermentas, Thermo Scientific). All primers used in this study are listed in **Supplementary Table S2**. DNA fragments were digested by the corresponding restriction enzymes (Fermentas, Thermo Scientific) and ligated with T4 DNA ligase (Fermentas, Thermo Scientific) according to the manufacturer’s instructions. Plasmid DNA was isolated using the GeneJET Plasmid Miniprep Kit (Fermentas, Thermo Scientific). DNA was transferred into *P. aeruginosa* cells either via electroporation using a Gene Pulser Xcell^TM^ electroporator (Bio-Rad) or via conjugation, as previously described (Crespo et al., 2015).

pETS191 and pETS192 plasmids were generated by applying PCR-based site-directed mutagenesis at the putative Anr/Dnr binding boxes of the P*nrdJ* and P*nrdD* promoter regions (TTGA^T^/_C_NNNN^A^/_G_TCAA, from the PRODORIC database^1^) and then cloning the resultant mutant promoters into pETS130-GFP plasmids. Anr/Dnr box mutagenesis was performed according to previously published procedures (Urban et al., 1997) using the following primers: for the P*nrdJ* promoter region, mutanrJ-up/mutanrJ-low as the inner primers and PnrdJ BamHI new-up/PnrdJ SmaI new-low as the outer primers; for the P*nrdD* promoter region, mutanrD-up/mutanrD-low as the inner primers and PnrdD-up/PnrdD new-low as the outer primers. The mutant fragments obtained from this process were cloned into pGEM-T Easy vectors, and the Anr/Dnr box mutation was verified via DNA sequencing. Finally, the fragments were digested with the corresponding restriction enzymes (BamHI/SmaI for P*nrdJ* and BamHI/ClaI for P*nrdD*) and cloned into pETS130-GFP plasmids.

For pETS193 generation, the *oprF* promoter region was amplified from *P. aeruginosa* PAO1 genomic DNA using the following primer pair: PoprFBHI-up/PoprFClaI-low. The amplicon (460 bp) was cloned into pGEM-T Easy vectors, verified via DNA sequencing, digested with BamHI/ClaI and cloned into pETS130-GFP plasmids.

For pETS195 generation, an amplicon containing the *dnr* promoter region and the full ORF (1128 bp) was amplified from *P. aeruginosa* PAO1 genomic DNA using the following primer pair: Pdnr-BHI up/Dnr-low. The amplicon was cloned into pGEM-T Easy vectors, verified via DNA sequencing, digested with BamHI/SalI and cloned into pUCP20T plasmids.

A *P. aeruginosa* Δ*nrdJ*Δ*nrdD* double mutant strain (ETS125) was constructed from the *P. aeruginosa* PAO1 *nrdD*::ΩTc; Tc^R^ (ETS103) strain (Sjoberg and Torrents, 2011) through the insertion of the gentamicin-resistance gene (*aacC1*) into the *nrdJ* gene using homologous recombination with the pETX100-Tlink vector, as previously described (Quenee et al., 2005). Briefly, two 400 bp areas surrounding the *nrdJ* gene were amplified via PCR with the following primer pairs: Jmut1HIIIup/Jmut2BIlw and Jmut3BIup/Jmut4SIlw. The two amplicons obtained were then cloned separately into pGEM-T Easy vectors. A plasmid containing both fragments was generated by BamHI/SacI digestion. The gentamicin resistance gene *aacC1* was obtained using *BamHI* digestion of pUCGmlox, and the corresponding cassette was ligated inside the two previous fragments. Next, the construct was cloned into the pEX100Tlink vector. The obtained plasmid pET100Tlink-*nrdJ*::ΩGm was transferred into the S17.1λ*pir* strain and conjugated to the *P. aeruginosa* ETS103 strain. Transformants were selected by plating them with tetracycline and gentamicin; 5% sucrose was added for *sacB-*mediated counterselection of the plasmids. The insertion of *aacC1* was screened via PCR with the primer pair Jmut1HIIIup/Jint-2-3lw and later confirmed via DNA sequencing.

### Quantitative Reverse Transcription PCR (qRT-PCR)

RNA from *P. aeruginosa* PAO1 cells (either planktonic or from a biofilm) was isolated with RNAprotect Bacterial Reagent (Qiagen), according the manufacturer’s instructions. RNA purification steps were carried out using the RNeasy Mini Kit (Qiagen), according the manufacturer’s instructions. DNase I (Turbo DNA-free, Applied Biosystems) was used to remove the remaining DNA, and RNA samples were subjected to PCR to verify the absence of DNA. For cDNA synthesis, RNA was quantified using a NanoDrop 1000 spectrophotometer (Thermo Scientific), and 0.5 μg of RNA was reverse transcribed with SuperScript III Reverse Transcriptase (Applied Biosystems) using the following primers for each gene: nrdATaqM2-low for *nrdA*, nrdJTaqM2-low for *nrdJa*, nrdDTaqM2-low for *nrdD* and gapTaqM-low for *gapA*. qRT-PCR quantification used nrdA-FAM, nrdJ-FAM, nrdD-FAM, and gap-FAM qRT-PCR probes (Crespo et al., 2015).

### Western Immunoblot Analysis

Western blotting was carried out as previously described (Sjoberg and Torrents, 2011), using anti-NrdJ (Agrisera, Sweden; and Thermo Fisher, USA) at a 1:1000 dilution. The detection of primary antibodies was performed using donkey anti-rabbit (Bio-Rad) horseradish peroxidase-conjugated secondary antibodies at a 1/50,000 dilution. The antibody-antigen complex was detected using the Amersham^TM^ ECL^TM^ Prime western blotting reagent (GE Healthcare), according the manufacturer’s protocol. Proteins were visualized and analyzed using an ImageQuant^TM^ LAS4000 mini system (GE Healthcare).

### Static and Continuous-Flow Biofilm Formation

To determine the biomass of static biofilms grown under aerobic conditions, cells were grown on 96-well plates (Nunclon Delta Surface, Thermo Scientific) in LB containing 0.2% glucose for 3 days at 37°C. Fully anaerobic static biofilms were grown in the same plates using LBN medium containing 0.2% glucose, and they were incubated inside GENbag ANAER (Biomerieux) devices. After the incubation period, the culture supernatant was removed, and both kinds of biofilm plates were washed three times with 1x phosphate buffered saline (PBS) to eliminate any remaining planktonic cells. Cells attached to the wells were then fixed with methanol and stained with 1% crystal violet (Cendra Mdel et al., 2012). After staining, excess crystal violet was eliminated with water, and 33% acetic acid was used to dissolve the remaining dye. Biofilm mass was finally determined as a function of the concentration of this dye based on the absorbance at 570 nm (*A*_570_).

Continuous-flow biofilms were cultured as previously described (Christensen et al., 1999; Baelo et al., 2015) with the following modifications. Biofilms were grown into three-channel flow cells made of Perspex [poly(methyl methacrylate), channel size 40 mm × 4 mm × 1 mm; DTU Systems Biology, Technical University of Denmark) covered with a n°1 24 mm × 50 mm glass coverslip (Deltalab, ref. D102450) which served as the biofilm substratum. Flow cells were supplied with LB broth supplemented with 0.2% glucose, pumped by a high precision multichannel peristaltic pump (Ismatec ISM 943, Idex). Flow cells were inoculated using a 1-ml syringe with a 26 G needle and kept static for 1 h. After this point, flow was initiated at a rate of 3 ml/channel/hour. After 5–6 days of growth, biofilms were analyzed through staining the formed biofilm with the LIVE/DEAD BacLight Bacterial Viability Kit (Molecular Probes, Life Science), according to the manufacturer’s instructions, and the biofilms were visualized with a Leica TCS SP5 confocal scanning laser microscope (CSLM; Leica Microsystems, Wetzlar, Germany). The excitation wavelength was 488 nm and the emission wavelength was 500 nm. Images were obtained using a 20×/0.70 air objective. Simulated fluorescence projections and sections were generated using ImageJ software, and COMSTAT 2 software was used to quantify the biomass and thickness of the biofilms (Weiss Nielsen et al., 2011).

### Green Fluorescent Protein Gene Reporter Assay

Promoters of the different RNR genes fused to GFP in pETS130-GFP plasmids were used to determine RNR gene expression [pETS134 (P*nrdA*::GFP), pETS180 (P*nrdJa*::GFP) and pETS136 (P*nrdD*::GFP)]. pETS191 (P*nrdJ*ΔAnr/Dnr-box::GFP) and pETS192 (P*nrdD*ΔAnr/Dnr-box::GFP) plasmids were used to evaluate the effect of an Anr/Dnr box mutation on *nrdJ* and *nrdD* expression, respectively. pETS193 (P*oprF*::GFP) plasmids were used as a control.

For liquid culture experiments, GFP fluorescence was measured in 96-well plates (Costar^®^ 96-Well Black Polystyrene Plate, Corning) on an Infinite 200 Pro Fluorescence Microplate Reader (Tecan), as previously described (Crespo et al., 2015). Briefly, three independent 1-ml samples of cells harboring the corresponding gene reporter assay plasmids grown to the mid-logarithmic phase (OD_550_) were collected and pelleted. Cells were fixed with 1 ml of a freshly prepared 1x PBS solution containing 2% formaldehyde and stored in the dark at 4°C. Three measurements were performed for each independent sample.

To determine gene expression during biofilm formation, experiments were performed on static biofilms formed in 96-well plates (Nunclon Delta Surface, Thermo Scientific) after the incubation of a liquid culture of the corresponding strain in LB containing 0.2% glucose at 37°C. After incubating the plate for a specific time (from 3 to 72 h), the culture supernatant was removed, and each well was washed three times with PBS to eliminate and remaining planktonic cells. The biofilm cells attached to the wells were then fixed with PBS containing 2% formaldehyde. Finally, fluorescence measurements were performed on an Infinite 200 Pro Fluorescence Microplate Reader (Tecan).

## Results

### Anaerobic RNR Classes Play an Important Role in *P. aeruginosa* Biofilm Formation

The *P. aeruginosa* genome encodes genes for three different ribonucleotide reductase (RNR) genes, two of them (class II and III) able to function enzymatically under anaerobic conditions, as previously described (Sjoberg and Torrents, 2011). The individual Δ*nrdJ* and Δ*nrdD* mutant strains showed a strong reduction in their anaerobic growth capacity (**Supplementary Table S3**). The Δ*nrdD* strain was able to grow under anaerobic conditions when supplemented with adenosylcobalamin or vitamin B_12_ to enhance class II RNR activity, agreeing with our previous report (Torrents et al., 2005; Sjoberg and Torrents, 2011). In this work, we generated a double class II (Δ*nrdJ*) and class III (Δ*nrdD*) RNR mutant (ETS125) that was unable to grow anaerobically (only growing to OD_550_ = 0.05 after a standard overnight anaerobic culture) and only was capable to grow under aerobic conditions (OD_550_ = 3.8). These growth patterns indicate the simultaneous need for class II and III RNRs in *P. aeruginosa* for anaerobic metabolism (**Supplementary Table S3**).

**FIGURE 1:**
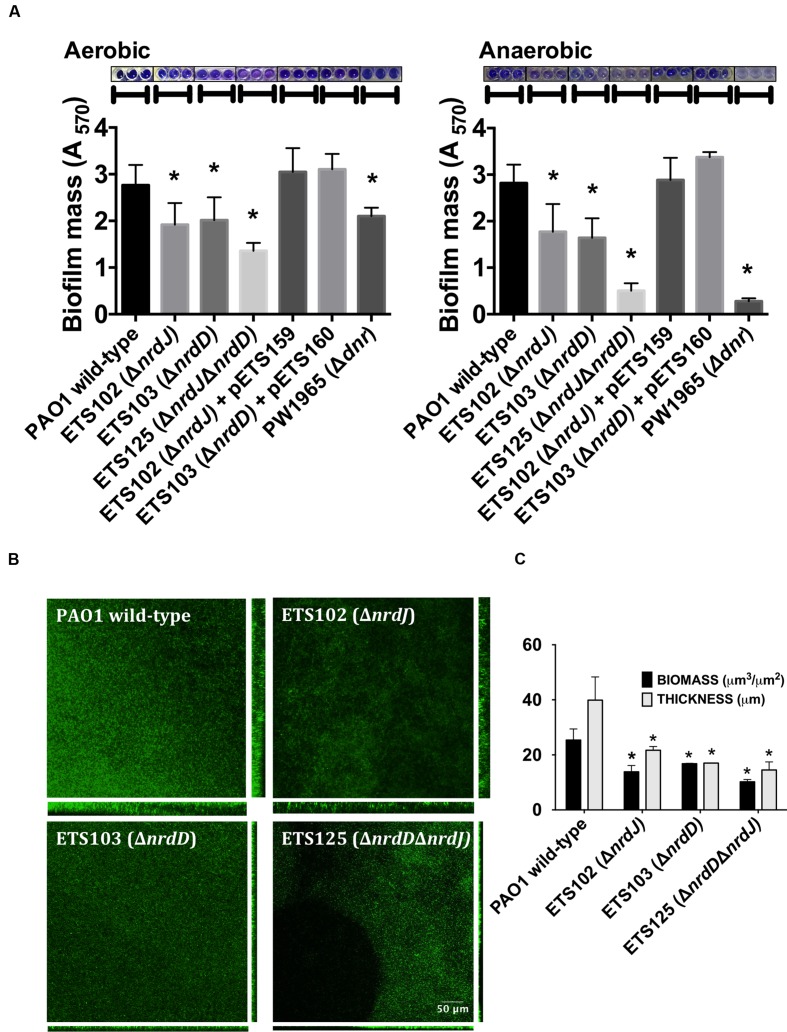
**Biofilm formation in *Pseudomonas aeruginosa* PAO1 wild-type and Ribonucleotide reductase (RNR) mutant strains.** **(A)** Initially aerobic and fully anaerobic static biofilm biomass quantification after growing at 37°C for 4 days. Each value is accompanied by the corresponding crystal violet-stained biofilm image. More than 20 replicates were performed in three independent experiments. The *nrdJab* and *nrdDG* genes cloned into pETS159 and pETS160 plasmids were used to complement *nrdJ* and *nrdD* deficiencies in ETS102 and ETS103 strains. The Δ*dnr* strain was included to compare RNR mutant strains with a strain defective in anaerobic metabolism. **(B)** Confocal laser scanning microscopy (CLSM) images of continuous-flow biofilms (sum of stack images) and their corresponding orthogonal views for *P. aeruginosa* PAO1 wild-type, ETS102 Δ*nrdJ*, ETS103 Δ*nrdD* and ETS125 Δ*nrdD*Δ*nrdJ* strains. **(C)** Quantification of total biomass (μm^3^/μm^2^) and average thickness (μm) for the biofilms from the previous continuous-flow experiments. Data are the average of three independent experiments. ^∗^Significantly different from the wild-type strain in an unpaired *t*-test (*P* < 0.05).

As anaerobic growth is needed to support full biofilm establishment (Stewart and Franklin, 2008), we explored the role of the different RNR classes in *P. aeruginosa* biofilm formation. The *P. aeruginosa* PAO1 wild-type strain, single Δ*nrdJ* (class II RNR) and Δ*nrdD* (class III RNR) isogenic mutant strains and double Δ*nrdJ*Δ*nrdD* mutant strain were assayed for their ability to form static biofilms under aerobic and anaerobic conditions (**Figure 1A**). The class I RNR mutation (Δ*nrdA*) strain is not viable and was not used in the current study (Sjoberg and Torrents, 2011). The results show that deficiencies in class II RNR activity (in ETS102 Δ*nrdJ* strain) and in class III RNR activity (in ETS103 Δ*nrdD* strain) resulted in decreased static biofilm formation under both aerobic and anaerobic initial conditions. Complementation of the mutation with a copy of the corresponding wild-type RNR gene (*nrdJ* or *nrdD* cloned into plasmids pEST159 and pETS160, respectively) returned biofilm formation to a level similar to that of the wild-type strain. The double Δ*nrdJ*Δ*nrdD* RNR mutant (ETS125) showed almost no biofilm formation, and the decrease was even stronger in anaerobic biofilm formation experiments, demonstrating the key role of anaerobic RNR activity in *P. aeruginosa* biofilm formation. However, our results suggest that anaerobic RNR activity is needed for biofilm formation even when the experiment is performed under aerobic conditions. A *P. aeruginosa* Δ*dnr* mutant strain (PW1965), unable to grow anaerobically, was used to compare the results from the RNR mutant with those from a strain unable to perform general anaerobic metabolism. Dnr is a transcriptional factor that regulates the expression of essential genes during *P. aeruginosa* anaerobic growth (Trunk et al., 2010). As expected, the PW1965 strain showed strong differences in biofilm formation when compared with the wild-type strain, even when the initial culture conditions were aerobic, and its ability to form biofilms resembled the ability shown by the ETS125 double RNR mutant strain.

To further corroborate our previous static biofilm formation experiments, we explored the importance of the different RNR classes in continuous-flow biofilm formation performed in flow cells. This technique better mimics the biofilms found in nature and specifically in the mucus plaques within the lungs of CF patients (Weiss Nielsen et al., 2011; Lebeaux et al., 2013). Biofilm cultures of different strains were cultivated under a continuous flow of LB medium over 6 days to obtain a robust and mature biofilm. The formed biofilms were then stained and visualized using confocal laser scanning microscopy (CLSM), as described in the section “Materials and Methods.”

**Figure 1B** shows the images obtained for the biofilms formed by the different strains that were evaluated and their corresponding orthogonal views. The thickness and total biomass values for each biofilm, estimated by COMSTAT software, are presented in **Figure 1C**. The biomass (μm^3^/μm^2^) and average thickness (μm) of the biofilms formed by all RNR mutant strains were decreased; biomass of the wild-type strain biofilm was 2.2, 1.8, and 2.7 times higher than the corresponding biomass of the anaerobic RNR mutant strains (ETS102 Δ*nrdJ*, ETS103 Δ*nrdD* and ETS125 Δ*nrdD*Δ*nrdJ*, respectively). The greatest thickness observed was in the *P. aeruginosa* wild-type strain biofilm (49.40 μm), while the different class II and III RNR mutant strains formed significantly thinner biofilms, with an average thickness of 24.84, 15.5, and 14.53 μm for ETS102 Δ*nrdJ*, ETS103 Δ*nrdD*, and ETS125 Δ*nrdD*Δ*nrdJ*, respectively. It is important to note that the *P. aeruginosa* double RNR class mutant (ETS125 Δ*nrdD*Δ*nrdJ*) grew in a discontinuous pattern and showed difficulties in attaching to the glass surface. These results confirm our previous observations in static biofilms, highlighting the importance of anaerobic RNRs in biofilm formation even when culture conditions are initially aerobic.

### RNR Enzymes Contribute to Proper Cell Division in a Biofilm

**FIGURE 2:**
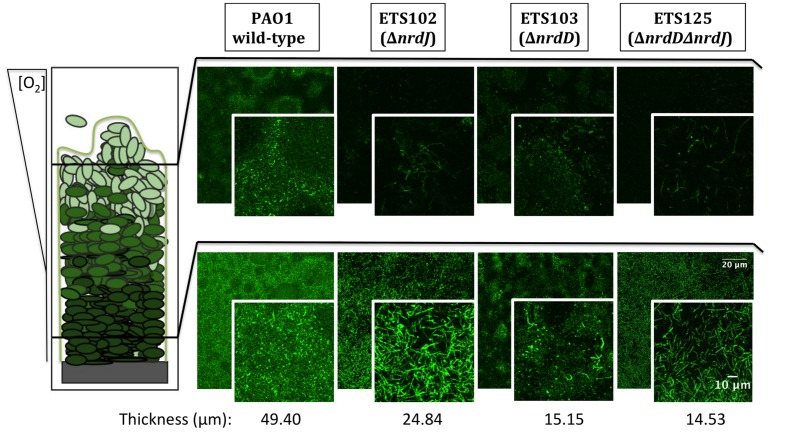
**Detailed microscopy observations of structured biofilms from *P. aeruginosa* PAO1 wild-type, ETS102, ETS103, and ETS125 strains.** On the left side, a scheme of the longitudinal structure of *P. aeruginosa* biofilm is represented, labeled with indications of the oxygen concentration along the biofilm (Stewart and Franklin, 2008). On the right side, CLSM images are shown, which were taken from the aerobic region of the biofilm (top part, superficial biofilm) and from the anaerobic region (bottom part, deeper in the biofilm structure). An internal panel with a magnification of a local area is shown for each image. The corresponding average thickness of each strain is representative of three independent experiments.

**Figure 2** shows the CLSM analysis of the longitudinal cell morphology in a structured biofilm formed by the different *P. aeruginosa* strains (PAO1 wild-type, ETS102 Δ*nrdJ*, ETS103 Δ*nrdD* and ETS125 Δ*nrdD*Δ*nrdJ*). As described previously, the different RNR mutant strains showed elongated morphologies during anaerobiosis (Lee et al., 2012). The *P. aeruginosa* wild-type cells showed a normal rod-shape cell morphology throughout the biofilm in both the aerobic and anaerobic regions (top and bottom segments of the biofilm, respectively). However, the *P. aeruginosa* ETS102 Δ*nrdJ* mutant strain showed significant cell elongation in both the top and the bottom parts of the biofilm structure, indicating some disturbances in cell growth and division, as was clearly demonstrated in previous planktonic anaerobic cultures (Yoon et al., 2011; Lee et al., 2012). Some *P. aeruginosa* ETS103 Δ*nrdD* cells also showed cell elongation but only in the bottom layer of the biofilm (anaerobic region), while rod-shaped cells were found in the upper region that were similar to the shapes of the wild-type strain. Finally, the *P. aeruginosa* double mutant (ETS125 Δ*nrdJ*Δ*nrdD*) exhibited cell elongated along the entire span of the biofilm, similar to the results seen in the II RNR mutant (ETS102 Δ*nrdJ*).

### During Biofilm Formation, Expression of the *nrdJ* and *nrdD* Genes Is Increased

Our previous results demonstrate the importance of class II and class III RNRs for anaerobic growth and biofilm formation in *P. aeruginosa* and show that these two processes are related as biofilm growth is characterized by a decrease oxygen tension that results in anaerobic conditions in the bottom regions of the structure (Werner et al., 2004; Stewart and Franklin, 2008). We hypothesized that the expression of class II and class III RNRs could be induced under these growing conditions. To explore this, we studied the induction of the different RNR genes using RT-PCR.

First, we explored the induction of RNR genes by comparing anaerobic growth with aerobic growth in liquid cultures at the stationary phase (**Table 1**). The results showed a strong increase in *nrdJa* and *nrdD* expression (85.2 and 110.6), while *nrdA* expression (2.1 times) was only slightly increased under anaerobic conditions.

**Table 1:** Relative expression of ribonucleotide reductase (RNR) genes based on real-time PCR.

	Differential expression (fold-change)		
	
	*nrdA*	*nrdJa*	*nrdD*
Planktonic anaerobic vs. Planktonic aerobic	2.1 ± 0.4	85.2 ± 5.0	110.6 ± 19.2
Biofilm aerobic vs. Planktonic aerobic	13.1 ± 6.2	1500 ± 150	128.2 ± 5.1
Biofilm aerobic vs. Planktonic anaerobic	2.4 ± 1.0	51.6 ± 7.3	-12.3 ± 1.6


*Fold change in *Pseudomonas aeruginosa* PAO1 *nrdA, nrdJa*, and *nrdD* transcription determined using real time PCR from 16-h-old planktonic cells grown aerobically or anaerobically and 4-day-old cells growing in biofilms. “Biofilm aerobic” refers to biofilms grown under initially aerobic conditions. The *gap* gene was used as an internal standard. The results shown represent the average of three independent experiments ± standard deviation.*

We also explored the effect of biofilm growth itself on RNR expression. To do this, we analyzed the RNA expression of each RNR class in aerobic planktonic cells (at the stationary phase) relative to the RNR expression in cells growing in aerobically made biofilms (a 4-day-old biofilm; **Table 1**) using RT-PCR. The results obtained in the *P. aeruginosa* wild-type strain showed significant differences in RNR expression between the two conditions: expression levels of *nrdA* showed a slight increase, but the expression of *nrdJa* and *nrdD* were both highly induced in the cells forming a robust biofilm relative to expression in the planktonic culture.

The induction of *nrdJa* and *nrdD* gene expression shown in biofilm formation and under anaerobic conditions could be due to control by factors related to anaerobic metabolism (i.e., factors acting in anaerobic cultures and in the anaerobic areas of biofilms) or/and due to specific biofilm-related factors. As a first approach to exploring this control, we examined the patterns of our previous RT-PCR results (**Table 1**). When comparing the results in the initially aerobic biofilm conditions with those of the anaerobic planktonic conditions, it is clear that *nrdJa* expression was highly increased during biofilm formation (1500 fold-change vs. 85), while *nrdD* expression was increased to a higher rate by factors related to anaerobic metabolism (almost same fold-change levels 110 vs. 128).

### Class II RNRs Are Transcriptionally Activated by a *dnr* Transcription Factor

To this point, we have demonstrated that class II and class III RNRs are of great importance for biofilm formation and that biofilm growth and an anaerobic environment strongly induce their expression. Therefore, the key transcriptional factors involved in *P. aeruginosa* anaerobic metabolism (Anr, Dnr, and NarL) were studied as putative transcriptional regulators for inducing RNR anaerobic expression (Arai, 2011; Arat et al., 2015).

The different transcription factors (Anr, Dnr, and NarL) are responsible for the regulation of different parts of the reduction chain in anaerobic respiration in *P. aeruginosa* [from NO_3_ to N_2_, through NO_2_ and NO (Trunk et al., 2010)]. Therefore, in the anaerobic transcriptional regulation study, *P. aeruginosa* was grown using KNO_3_ and GSNO (a NO donor) as final electron acceptors to obtain more information about which transcriptional regulator might be involved in the RNR transcriptional regulation under anaerobic conditions.

**FIGURE 3:**
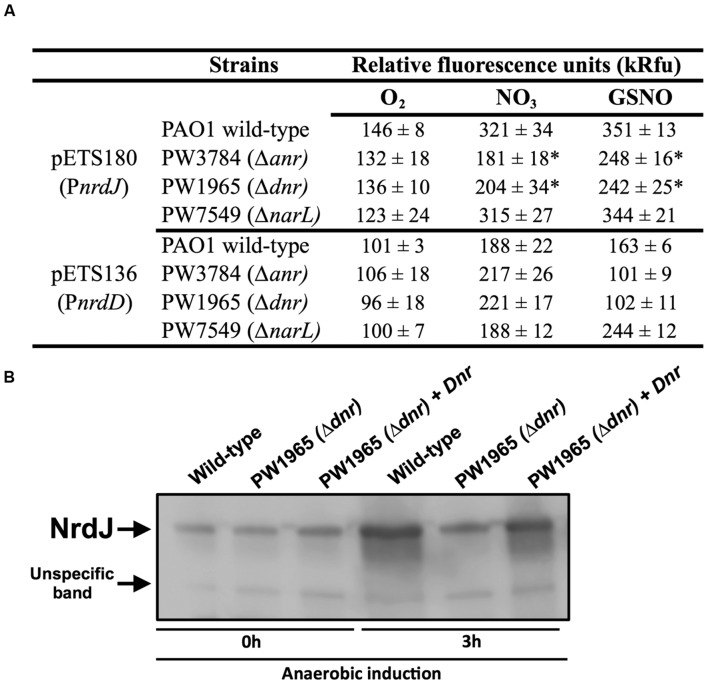
**Transcriptional regulation of *nrdJ* and *nrdD* during anaerobic growth.** **(A)** Transcriptional expression of *nrdJ* (pETS180) and *nrdD* (pETS136) promoters in *P. aeruginosa* wild-type, PW3785 Δanr, PW1965 Δdnr and PW7549 ΔnarL strains. Cells were grown aerobically to a mid-logarithmic phase (A550 0.5) and then were grown anaerobically (with NO3 or GSNO as electron acceptors) for 3 h to induce anaerobic metabolism. Values are the means SD from more than three independent experiments. ^∗^Significantly different from the *P. aeruginosa* wild-type strain in an unpaired *t*-test (*p*-value 0.05). kRfu 1000 relative fluorescence units. **(B)** NrdJ protein expression analysis in PAO1 wild-type, PW1965 Δ*dnr* and PW1965 Δ*dnr* + pETS195 (*dnr* complementation plasmid) via western blot analysis performed at a mid-logarithmic phase after 0 h or 3 h of anaerobic induction. A representative blot of three independent western blot analyses is shown. An unknown, unspecific band that is present at an almost constant intensity in all samples is shown in the blot and served as a loading control.

Transcriptional fusions of the *nrdJ* and *nrdD* promoter regions to GFP (present in the pETS180 and pETS136 plasmids, respectively) were transformed in different *P. aeruginosa* strains (PAO1, PW3784 Δ*anr*, PW1965 Δ*dnr* and PW7549 Δ*narL*) and used for gene reporter assays (see Materials and Methods). As seen in **Figure 3A**, the results show an increased expression of both *nrdJ* and *nrdD* under anaerobic growth in the presence of both 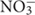 (321 and 188, respectively) and GSNO (351 and 163, respectively) compared to the expression during aerobic growth (146 and 101, respectively).

Comparing *nrdJ* expression between the *P. aeruginosa anr*, *dnr*, and *narL* knockout mutant strains and the wild-type strain (**Figure 3A**), we identified a reduced anaerobic induction of *nrdJ* expression in the Δ*anr* (PW3784) and Δ*dnr* (PW1965) mutant strains compared with the values of the wild-type strain. No effect was observed on *nrdJ* transcription when the *narL* gene was mutated. Our results show the dependence of the anaerobic induction of *nrdJ* gene expression on Anr and Dnr transcriptional regulators. As the effect is shown when any of these two genes are mutated and Dnr is controlled by Anr (which acts early in the regulatory chain of anaerobic metabolism), Dnr was considered the most likely candidate for being responsible for regulating *nrdJ.*

This control of *nrdJ* expression by Dnr was later verified at the protein level using a western blotting assay (**Figure 3B**). Although no differences were found in the amount of NrdJ protein between *P. aeruginosa* wild-type and PW1965 Δ*dnr* mutant strains when measured during aerobic growth, 3 h of anaerobic metabolism induced a strong reduction in NrdJ levels in the Δ*dnr* strain relative to expression in the wild-type cells. This effect was reverted back to near wild-type levels by Dnr complementation using the pETS195 complementation plasmid.

We failed to identify any regulation on *nrdD* expression by anaerobiosis-related Anr, Dnr, or NarL factors, as demonstrated in our results (**Figure 3A**).

**FIGURE 4:**
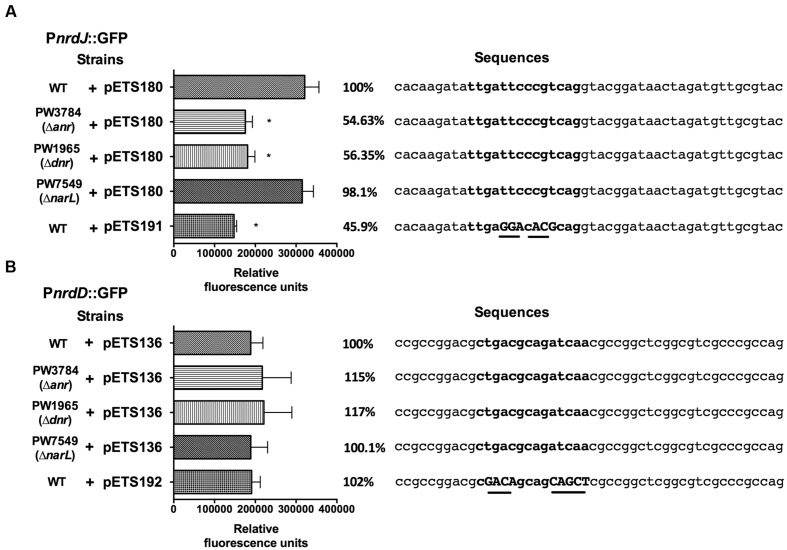
***nrdJ* and *nrdD* expression in *P. aeruginosa* wild-type, PW3784 Δ*anr*, PW1965 Δ*dnr* and PW7549 Δ*narL* strains.** GFP fluorescence of **(A)** P*nrdJ* and P*nrdJ* ΔAnr/Dnr-box and **(B)** of P*nrdD* and P*nrdD* ΔAnr/Dnr-box measured in the wild-type, PW3784 Δ*anr*, PW1965 Δ*dnr* and PW7549 Δ*narL* strains at a mid-logarithmic phase after 3 h of anaerobic induction. A fragment of the sequence of the corresponding promoter regions surrounding the putative Anr/Dnr-box is added at the right, and the sequence of the box is indicated in bold letters. In the mutant Anr/Dnr-boxes, mutated nucleotides are indicated in capital letters and underlined. The Anr/Dnr box is centered at -84 and -98 bp from the translation start site of the *nrdJ* and *nrdD* genes, respectively. ^∗^Significantly different from the wild-type strain in an unpaired *t*-test (*P* < 0.05). Error bars represent the standard deviation for three independent experiments.

To determine if the anaerobiosis-related transcriptional factors bind specifically on the RNR promoters, a bioinformatic search of putative Anr-Dnr binding sites was performed on the P*nrdJ* and P*nrdD* promoter regions. One Anr/Dnr-box was identified on both P*nrdJ* and P*nrdD* promoters (see Materials and Methods) according to the TTGA^T^/_C_NNNN^A^/_G_TCAA consensus present in PRODORIC database. The putative Anr/Dnr boxes identified are shown in **Figure 4**.

To confirm the binding of Anr/Dnr to the promoters, we specifically mutated the essential nucleotides of the putative Anr/Dnr-boxes identified, fused the mutant promoters to GFP and constructed plasmids for gene reporter assays (pETS191 for the *nrdJ* promoter and pETS192 for the *nrdD* promoter). In the corresponding assay, P*nrdJ* expression decreased when the Anr/Dnr-box was mutated. Moreover, the ΔAnr/Dnr-box P*nrdJ* (pETS191) expression was similar to that found in Δ*anr* and Δ*dnr* mutant strains (**Figure 4A**). However, no significant results were obtained when mutating the P*nrdD* Anr/Dnr-box (**Figure 4B**).

### The Presence of Anaerobic Environments in the Biofilm Increases *nrdJ* Expression Through *dnr* Activation

Our previous results demonstrate that class II and class III RNRs are of great importance for biofilm formation and anaerobic growth in *P. aeruginosa* and that their expression is specifically induced under these conditions. As the induction of anaerobic metabolism increases as biofilm growth advances, we expected to detect a progressive induction of the expression of both RNRs during biofilm establishment and maturation.

To determine this, a GFP-based gene reporter assay was performed on a static biofilm culture over time. The expression of wild-type P*nrdA*, *PnrdJ*, and P*nrdD* was determined together with that of the mutant versions of P*nrdJ* and P*nrdD* (carrying mutant Anr/Dnr-boxes); a promoterless GFP plasmid (pETS130) was used as a negative control, and the *oprF* promoter (P*oprF*) was used as a positive control for anaerobic induction. OprF is a membrane protein that has its highest expression under anaerobic conditions, and it can be used as a marker of infection in a CF patient’s lung or sputum (Yoon et al., 2002; Eichner et al., 2014).

**FIGURE 5:**
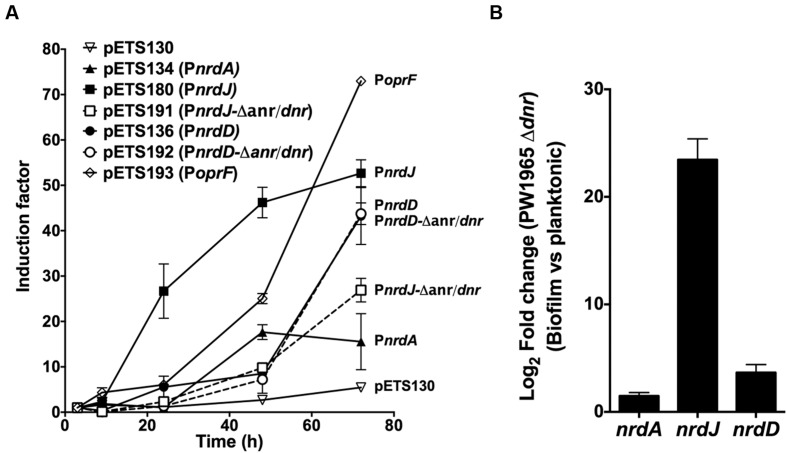
**Regulation of RNR expression during biofilm formation.** **(A)** GFP-based gene reporter assay of *P. aeruginosa* cells growing as a static biofilm. The induction factor is expressed as the quotient of the fluorescence units measured from one strain at one point in time relative to the corresponding value measured at the first time point (3 h of culture). Each strain was monitored between 3 and 72 h of biofilm growth. Wild-type RNR promoters (P*nrdA*, P*nrdJ*, and P*nrdD*) are represented in continuous lines, and mutant RNR promoters (P*nrdJ* and P*nrdD* carrying a mutant version of the putative Anr/Dnr-box identified) are plotted in dotted lines. A promoterless GFP in pETS130 plasmids was used as a negative control, and the P*oprF* promoter in pETS193 plasmids was used as a positive control for anaerobic induction of gene expression. **(B)** Fold change in *P. aeruginosa* PW1965 Δ*dnr* P*nrdA*, P*nrdJ*, and P*nrdD* promoter transcription was determined through real time PCR in 4-day-old cells grown as a static biofilm and compared with transcription in 16-h-old planktonic cells, both of which were cultured under aerobic conditions. The *gap* gene was used as an internal standard. The results shown represent the mean of three independent experiments ± standard deviation.

As expected (**Figure 5A**), P*oprF* expression increased greatly during mature biofilm development, demonstrating the progressive establishment of anaerobic conditions in the deep layers of the biofilm structure. Simultaneously, the P*nrdJ* and P*nrdD* promoter expression increased, although P*nrdD* expression increased only in the later stages when a mature and robust biofilm was formed. Mutating the Anr/Dnr-box severely reduced P*nrdJ* expression, while the anaerobic induction of P*nrdD* remained unaffected.

As a *P. aeruginosa* Dnr deficient strain is still able to grow as a biofilm, we further explored how Dnr controls RNR expression during biofilm formation by comparing *nrdA*, *nrdJ* and *nrdD* transcription in planktonic cells with that in biofilm-forming cells using the PW1965 Δ*dnr* mutant strain. Comparing the results obtained (**Figure 5B**) with those from a *P. aeruginosa* wild-type biofilm vs. planktonic comparison (**Table 1**), we can see that when mutating the Dnr gene, the induction of *nrdJ* expression in biofilms becomes severely reduced but not completely abolished. Surprisingly, we also noticed that the induction of *nrdD* expression was also strongly reduced in the PW1965 Δ*dnr* strain, which is in contrast with what was observed in the gene reporter assays (**Figures 3A** and **4B**).

## Discussion

*Pseudomonas aeruginosa* is well known for its genetic diversity. It has a relatively large genome (6.3 Mb) for a bacterium, and contains a large number of genes involved in different metabolic activities, which might contribute to the environmental adaptability of this bacterium. Its ability to grow in the absence of oxygen using nitrates or other forms of oxidized nitrogen as electron acceptors is an important example of *P. aeruginosa’s* anaerobic growth capacity (Trunk et al., 2010; Arat et al., 2015), which opens up a wide range of environments in which *P. aeruginosa* can grow.

Such anaerobic environments are present in a mature biofilm, in which different nutrient gradients and differential physical properties appear. Previous reports have highlighted the oxygen concentration heterogeneity in biofilms using microelectrodes, and have described the oxygen diffusion profiles in continuous biofilms (Werner et al., 2004). The oxygen concentration throughout the biofilm is thus a crucial parameter for bacterial growth in a mature biofilm (Stewart and Franklin, 2008) and strongly defines its morphogenesis and final structure (Dietrich et al., 2013; Kempes et al., 2014; Okegbe et al., 2014). Metabolites and oxygen easily diffuse in the outer layers of the biofilm; however, the free oxygen concentration becomes reduced in lower layers, resulting in strict anaerobic conditions in the depths of the mature biofilm. The three ribonucleotide reductase classes encoded by *P. aeruginosa* (class Ia, encoded in *nrdA* and *nrdB*; class II, encoded in *nrdJa* and *nrdJb*; and class III, encoded in *nrdD* and *nrdG*) are likely to increase the capacity of this bacterium to grow in the different environments generated throughout biofilms (Torrents et al., 2005; Sjoberg and Torrents, 2011).

Class Ia activity is strictly oxygen dependent, while class III is oxygen sensitive and can only function under strict anaerobic conditions. Class II is oxygen independent but needs vitamin B_12_ (*S*-adenosylcobalamin) for the completion of its catalytic cycle (Torrents et al., 2005). In accordance of these different levels of oxygen dependence, we hypothesized that all three RNR classes would have a predominant role in the progressively deeper layers of the biofilm structure, with class II and class III RNRs essential for anaerobic growth and therefore for the establishment of fully mature biofilms.

The most basic study was performed to analyze the differential ability of Δ*nrdJ* and Δ*nrdD* mutant strains and a Δ*nrdJ*Δ*nrdD* double mutant strain to grow in aerobic and anaerobic liquid cultures. The large reduction in anaerobic growth found after altering class II or class III RNRs highlights the importance of both RNR classes for anaerobic growth (**Supplementary Table S3**). In addition, the ability of class II RNRs alone to sustain anaerobic growth when the culture was supplemented with exogenous *S*-adenosylcobalamin suggests that class II RNRs can theoretically synthesize enough dNTPs to maintain normal growth rates, with *S*-adenosylcobalamin levels under anaerobic conditions being the limiting step.

The next step was to study how these same effects act on the natural formation of the anaerobic environments that appear during biofilm formation. Static biofilm formation was severely diminished when class II or class III RNRs were mutated (**Figure 1A**). This effect was higher when biofilms were built directly under anaerobic conditions but was also present under aerobic conditions. We associated this effect to the formation of anaerobic microenvironments in the biofilm depths that will undoubtedly occur if biofilms grow thick enough, and this was demonstrated by the analogous effect observed when mutating the *dnr* gene, which is one of the main transcriptional regulators of anaerobic metabolism (Schreiber et al., 2007). In this case, the impaired anaerobic metabolism implies that biofilm biomass will be reduced even when conditions are initially aerobic.

As static biofilm formation in microplates can be considered an artificial lab condition, we also studied the effect of class II and class III RNR alterations on continuous-flow biofilm formation, a technique that is thought to better the mimic biofilms present in nature and in clinically relevant cases, such as lung infections in CF patients (Weiss Nielsen et al., 2011; Lebeaux et al., 2013). Agreeing with our previous results, both biofilm biomass and thickness were considerably reduced when mutating the class II and/or class III RNRs (**Figures 1B,C**). The structure of the so-formed biofilm also changed compared with that of the wild-type biofilm. It is particularly important that in the Δ*nrdJ* Δ*nrdD* double mutant strain, a growth pattern of discontinuous patches appeared, showing the dependence on aeration of this strain.

**FIGURE 6:**
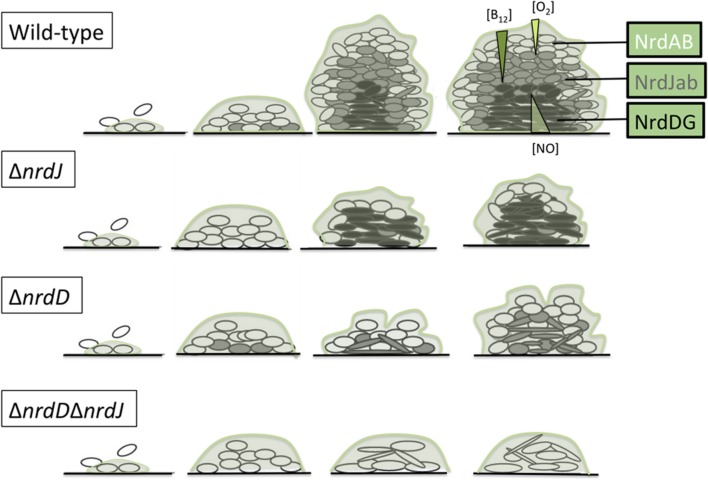
**Model of ribonucleotide reductases expression during *P. aeruginosa* biofilm formation.** Oxygen concentration is progressively reduced throughout the biofilm structure in a well-established gradient (Xu et al., 1998; Stewart and Franklin, 2008). Vitamin B_12_ and NO gradient concentrations also presented here are hypothetical (see the Discussion section).

All these data can be incorporated into a model in which the biofilm is considered a set of layers where the free oxygen concentration is progressively reduced with depth (**Figure 6**). Interestingly, vitamin B_12_ can only be synthesized under aerobic conditions (Lee et al., 2012); to our knowledge the diffusion properties of vitamin B_12_ in the biofilm have never been formally determined, but we can expect it to be gradually diffused throughout the biofilm layers and actively consumed when crossing them, therefore the deeper layers would not only be anaerobic but also limited in adenosylcobalamin. Therefore, at the top of the biofilm, class I RNRs would be the main enzyme responsible for dNTP synthesis, while class II RNRs would gain more importance in the middle layers (characterized by reduced oxygen levels but within the range of vitamin B_12_ diffusion) and class III RNRs would support growth in the lower layers as it does not depend on oxygen or metabolite diffusion from the outer regions.

Additionally, we studied the cell morphology in the different layers of the biofilm. A cell elongation phenotype is associated with impaired cell division, which can be triggered by depletion of the dNTP pool when RNR metabolism is affected. According to our model, the Δ*nrdJ* mutant strain and the Δ*nrdJ*Δ*nrdD* double mutant strain showed elongated cells throughout almost the entire biofilm depth, while the Δ*nrdD* strain only presented elongation in the lower layers (**Figure 2**). These results must be interpreted by also taking into account the fact that reductions in biofilm biomass and thickness were also happening, so the thin layer in which class the III RNR mutation seems to affect cell morphology means only that strictly anaerobic areas were unable to form.

Given the importance of class II and class III RNRs for anaerobic growth and biofilm formation, we expected an up-regulation of these enzymes under these conditions. It is known that as much as half of the *P. aeruginosa* genome is differentially expressed during biofilm development, including many genes involved in anaerobic metabolism, which are up-regulated in mature biofilms (Waite et al., 2006). Some studies have highlighted that the differential gene expression of class II RNRs depends on levels of oxygenation and have shown a 3.2-fold up-regulation under anaerobic conditions compared with expression during aerobiosis (Filiatrault et al., 2005). *nrdJ* up-regulation has also been noticed in anaerobic sputum (Palmer et al., 2007), and NrdJ and NrdD proteins were also identified to have an increased concentration under anaerobic conditions (Wu et al., 2005).

In agreement with these observations, we observed a large increase in *nrdJ* and *nrdD* mRNA levels under anaerobic conditions (compared with aerobiosis) and in biofilm-forming cells (compared with planktonic cells; **Table 1**). These results imply the existence of a direct or indirect mechanism to activate *nrdJ* and *nrdD* transcription as a result of anaerobic metabolism and/or due to specific biofilm-related factors. The comparison between the expression in initially aerobic biofilm cells and in anaerobic planktonic cells shows that *nrdD* transcription was mainly activated by anaerobiosis, while *nrdJ* expression levels appeared to also be regulated by specific biofilm factors, as *nrdJ* induction in the biofilm (where only some anaerobic and microaerophilic areas are present) is higher than in fully anaerobic planktonic cultures.

To sustain anaerobic metabolism, *P. aeruginosa* uses NO_3_ or other more oxidized forms of nitrogen (NO_2_, NO) as final electron acceptors for anaerobic respiration: the final product of the full chain of reductions is molecular nitrogen (N_2_; Schreiber et al., 2007). Anr acts as a general regulator of all anaerobic metabolism, activating the transcription of all metabolic enzymes thought to be involved in the pathway and that of the more specific regulators *dnr* and *narL*. NarL and Dnr transcription factors are in turn responsible for the control of the enzymes acting in the first reduction (from NO_3_ to NO_2_) and in the whole pathway, respectively. When analyzing the effects of mutations of these transcription factors on the RNR expression levels measured in a gene reporter assay, we observed a strong reduction in the anaerobic induction of *nrdJ* expression in the Δ*dnr* and Δ*anr* mutant strains, while no effect was observed when mutating the *narL* gene, and *nrdD* expression was not altered (**Figure 3**).

Therefore, we suggest that regulation by Anr/Dnr is partially responsible for class II RNR anaerobic induction. If Anr is active in the upper part of the regulation cascade, a simple transcriptional activation by Dnr would be the easiest explanation for the results obtained. Furthermore, as *nrdJ* expression was increased when GSNO, as an NO donor, was used as an electron acceptor, and NO levels affect the denitrification process by modulating Dnr regulation (Van Alst et al., 2007; Castiglione et al., 2009), these findings support the hypothesis of transcriptional control of *nrdJ* expression by Dnr. According to the biofilm reaction-diffusion theory (Stewart, 2003) we hypothesize that NO, described to be the main metabolite accumulated as a consequence of anaerobic metabolisms (Ye et al., 1994), should see its concentration increased in the lower layers, enhancing the effect of Dnr regulation (see **Figure 6**).

The genes belonging to the Anr/Dnr regulons are associated with Anr and Dnr binding boxes (Trunk et al., 2010), although the binding sites are still not well determined and more studies are needed to distinguish between them. Surprisingly, we identified a putative Anr/Dnr binding box in both class II and class III RNR promoters (P*nrdJ* and P*nrdD*; **Figure 4**). In our gene reporter assays, we determined that the mutation of the putative Anr/Dnr box in P*nrdJ* dramatically reduced the anaerobic induction of class II RNR expression (resembling the effect of *dnr* or *anr* gene mutation), while mutating the putative Anr/Dnr box in P*nrdD* had no significant effect.

According to these results, we can assume that under anaerobic conditions or in the anaerobic and microaerophilic environments generated during biofilm formation, NrdJ activity is essential for proper growth and that it is activated under these conditions by Dnr or Anr/Dnr via direct binding with its promoter. However, further studies are needed to determine if there are other specific biofilm-related factors activating NrdJ transcription and to define the mechanism for class III RNR anaerobic induction. This could be due to other factors that have not yet been studied, or it could even be related to Anr/Dnr pathways (as suggested the putative box found in the promoter) that may only be detectable under specific conditions that have not yet been tested.

Integrating our experiments on the effects of RNR mutation on biofilm formation and on RNR regulation in biofilm growth and under anaerobic conditions, we performed a gene reporter assay during biofilm formation, which supported our model: as the *P. aeruginosa* PAO1 wild-type biofilm structure matured, anaerobic areas were generated (as defined by the induction of the control promoter P*oprF*) and P*nrdJ* and P*nrdD* were consequently induced (**Figure 5A**). Again, mutating the putative Anr/Dnr boxes reduced class II RNR induction and had no effect on class III RNRs. However, analyzing the difference in expression in a PAO1 Δ*dnr* mutant strain between biofilm forming cells and planktonic cells, we not only observed a reduced anaerobic induction of class II RNRs but also, surprisingly, a considerably reduced induction of class III RNRs (**Figure 5B**), reinforcing the hypothesis that there could be an as-yet-undefined direct or indirect mechanisms by which Anr/Dnr controls *PnrdD* expression.

The model of a *P. aeruginosa* biofilm as a set of layers with different RNR expression profiles that are determined by oxygen concentration and B_12_ diffusion gradients and by cells with specific genetic regulation to support the differential RNR activities is of great importance for our understanding of this particular growth pattern. These results could play an important role in understanding the virulence of bacterial biofilms as it has been shown that the growth conditions in the lungs of CF patients include oxygen-limited growth and anaerobic environments (Schobert and Jahn, 2010) and that susceptibility to antibiotics in biofilms is modulated by limited oxygen availability (Borriello et al., 2006).

## Author Contributions

AC, LP, and ET designed the study. AC, LP, and JA performed the experiments. All authors analyzed the data, wrote the paper, read and approved the final version.

## Conflict of Interest Statement

The authors declare that the research was conducted in the absence of any commercial or financial relationships that could be construed as a potential conflict of interest.

## Footnotes

1 http://www.prodoric.de/vfp/
