# Supplementary material for: Pseudomonas aeruginosa Exhibits Deficient Biofilm Formation in the Absence of Class II and III Ribonucleotide Reductases Due to Hindered Anaerobic Growth
Source: Front Microbiol. 2016 May 9;7:688. doi: 10.3389/fmicb.2016.00688 (PMC4860495; doi:10.3389/fmicb.2016.00688)
Supplement: Supplementary file 1 [file Data_Sheet_1.PDF]

frontiers

Supplementary Material

Pseudomonas aeruginosa exhibits deficient biofilm formation in the absence of specifically activated class II and III ribonucleotide reductases

Anna Crespo, Lucas Pedraz, Josep Astola, and Eduard Torrents*

*** Correspondence:** Dr. Eduard Torrents, Bacterial Infections and Antimicrobial Therapies group, Institute for Bioengineering of Catalonia (IBEC), Baldiri Reixac 15-21, 08028, Barcelona, Spain; e-mail: etorrents@ibecbarcelona.eu

1 Supplementary Tables

- **Supplementary Table S1. Bacterial strains and plasmids used in this study.**
- **Supplementary Table S2. Primers and probes used in this study.**
- **Supplementary Table S3. Growth of *P. aeruginosa* wild-type and mutant strains under aerobic and anaerobic conditions.**

Supplementary Table S1. Bacterial strains and plasmids used in this study.

Strain or plasmid	Description	Source
**Plasmids**		
pGEM-T easy	A/T cloning vecto;, Amp^R	Promega
pUCP20T	Broad-host-range vector; Amp^R	(West et al., 1994)
pUCGmlox	pUC18-based vector containing the lox flanked aacC1 gene; Amp^R, Gm^R	(Quenee et al., 2005)
pEX100Tlink	*Pseudomonas* suicide vector pEX100T with a MCS, *sacB, oriT;* Amp^R	(Quenee et al., 2005)
pETS130-GFP	Broad host range, promoterless GFP; Gm^R	(Sjoberg and Torrents, 2011)
pETS134	pETS130 derivative carrying *nrdA* promoter; Gm^R	(Sjoberg and Torrents, 2011)
pETS136	pETS130 derivative carrying *nrdD* promoter; Gm^R	(Sjoberg and Torrents, 2011)
pETS159	pBBR1 derivative carrying *nrdJab* operon; Gm^R	(Sjoberg and Torrents, 2011)
pETS160	pBBR1 derivative carrying *nrdDG* operon; Gm^R	(Sjoberg and Torrents, 2011)
pETS180	pETS130 derivative carrying *nrdJ* promoter; Gm^R	(Crespo et al., 2015)
pETS191	pETS130 derivative carrying mutant Anr/Dnr box in *nrdJ* promoter; Gm^R	This work
pETS192	pETS130 derivative carrying mutant Anr/Dnr box in *nrdD* promoter; Gm^R	This work
pETS193	pETS130 derivative carrying *oprF* promoter; Gm^R	This work
pETS195	pUCP20T derivative carrying *dnr* gene; Amp^R	This work

pETS196	pET100Tlink-*nrdJ*::ΩGm	This work
**Strains**		
E. coli		
DH5α	*recA1 endA1 hsdR17 supE44 thi-1 relA1 Δ(lacZYA-argF)U169 deoR Φ80dlacZM15*	Laboratory stock
S17.1λ*pir*	*recA thi pro hsdR- M+RP4::2-Tc::Mu::Km Tn7 Tpr Smr Xpir*	(de Lorenzo et al., 1993)
P. aeruginosa		
PAO1	Wild-type (ATCC 15692 / CECT 4122)- Spanish Type Culture Collection	Lab strain
PW3784	*P. aeruginosa* PAO1 *anr*::*IS*l*acZ*/hah; Tc^R	(Jacobs et al., 2003)
PW1965	*P. aeruginosa* PAO1 *dnr*::*IS*l*acZ*/hah; Tc^R	(Jacobs et al., 2003)
PW7549	*P. aeruginosa* PAO1 *narL*::*ISlacZ*/hah; Tc^R	(Jacobs et al., 2003)
ETS102	*P. aeruginosa* PAO1 *nrdJ*:: ΩTc; Tc^R	(Sjoberg and Torrents, 2011)
ETS103	*P. aeruginosa* PAO1 *nrdD*::ΩTc; Tc^R	(Sjoberg and Torrents, 2011)
ETS125	*P. aeruginosa* PAO1 *nrdD*::ΩTc; Tc^R, *nrdJ*::ΩGm; Gm^R	This work

Supplementary Table S2. Primers and probes used in this study.

Name	Sequence (5'→3')	Application
M13-dir	GTTTTCCCAGTCACGAC	Check-Cloning
M13-rev	CAGGAAACAGCTATGACC	Check-Cloning
pUCP20T-up	CCTCTTCGCTATTACGCCAG	Cloning
pUCP20T-low	TCCGGCTCGTATGTTGTGTG	Cloning
pBBR1-up	CATCGCAGTCGGCCTATTGG	Cloning
pBBR1-low	CACTTTATGCTTCCGGCTCG	Cloning
PnrdA-up	AGGATCCGAATTCTTGCTCCACACAGCCTC	Cloning
PnrdA-low	ACCCGGGTTCTCGCGTGTGGTGTCG	Cloning
PnrdJ BamHI new-up	GGATCCCGCGCCCAGCTGAAGGCC	*PnrdJ* promoter cloning
PnrdJ SmaI new-low	AACCCGGGGACTGCGTTGCGTCTGTC	*PnrdJ* promoter cloning
PnrdD-up	AGGATCCGAATTCGCCCGCCTCGCCCAGG	*PnrdD* promoter cloning
PnrdD new-low	AATCGATCAGGGTGGCCGGCCAGGTAG	*PnrdD* promoter cloning
nrdATaqM2-low	TGTTCATGTCGTGGGTACG	qRT-PCR
nrdJTaqM2-low	GTAAACACCCGCACCACTTC	qRT-PCR
nrdDTaqM2-low	CCGAGTTGAGGAAGTTCTGG	qRT-PCR
gapTaqM-low	GAGGTTCTGGTCGTTGGT	qRT-PCR
nrdA-FAM	CTGGCACCTGGACATC	qRT-PCR probe
nrdJ-FAM	TCGGCTCGGTCAACCT	qRT-PCR probe
nrdD-FAM	CCCGACCTACAACATC	qRT-PCR probe
gap-FAM	CCTGCACCACCAACTG	qRT-PCR probe

mutanrJ-up	TATTGAGGACACGCAGGTACGGA	Mutation of Anr box in P*nrdJ*
mutanrJ-low	TCCGTACCTGCGTGTCCTCAATA	Mutation of Anr box in P*nrdJ*
mutanrD-up	GACGCGACAGCAGCAGCTCGCCGGC	Mutation of Anr box in P*nrdD*
mutanrD-low	GCCGGCGAGCTGCTGCTGTCGCGTC	Mutation of Anr box in P*nrdD*
Jmut1HIIIup	AAAGCTTCCCGTCAGGTACGGATAAC	*nrdJ* gene mutation
Jmut2BIlw	AAAAGGATCCATGGAGTCCTGGATGGTCC	*nrdJ* gene mutation
Jmut3BIup	AAAAGGATCCTATTACGGCAAGTACTGAGG	*nrdJ* gene mutation
Jmut4SIlw	AGAGCTCGACAAGGAAGGTGCAGTC	*nrdJ* gene mutation
Jint-2-3lw	TAGATGTCCATGAACGACAGC	checking *nrdJ* gene mutation
PoprFBHI-up	GGATCCCAACGAGTGCATCACG	P*oprF* promoter cloning
PoprFClaI-low	ATCGATGGTGTTCTTCAGTTTCAT	*PoprF* promoter cloning
Pdnr-BHI-up	GGATCCACGGCAGATGCACT	*dnr* cloning for complementation
Dnr-low	ATCACTCGAAGCACTCCAGGC	*dnr* cloning for complementation

Supplementary Table S3. Growth of *P. aeruginosa* wild-type and mutant strains under aerobic and anaerobic conditions. Δ*nrdJ*, Δ*nrdD*, Δ*nrdD*Δ*nrdJ* and wild-type PAO1 strains were grown for 16 h under aerobic and anaerobic conditions in LB and LBN, respectively. Bacterial growth was measured by reading the optical density at 550 (OD_{550}). Vitamin B_{12} was added when necessary at a concentration of 1 μg/mL. Final OD_{550} values are listed in the table.

		**OD_{550}**			
	**B_{12}**	**PAO1 wild-type**	**ETS102 Δ*nrdJ***	**ETS103 Δ*nrdD***	**ETS125 Δ*nrdD*Δ*nrdJ***
**Aerobic**	**-**	4.00	3.70	3.90	3.80
	+	3.90	3.80	3.87	3.76
**Anaerobic**	**-**	1.58	0.13	0.17	0.05
	+	1.91	0.13	2.00	0.07
